# Crisis responses, opportunity, and public authority during Covid‐19's first wave in Uganda, the Democratic Republic of Congo, and South Sudan

**DOI:** 10.1111/disa.12513

**Published:** 2021-11-29

**Authors:** Tom Kirk, Duncan Green, Tim Allen, Tatiana Carayannis, José Bazonzi, José Ndala, Patrycja Stys, Papy Muzuri, Aymar Nyenyezi, Koen Vlassenroot, Abraham Diing Akoi Nyuon, Anna Macdonald, Arthur Owor, Liz Storer, Joseph Okello, Julian Hopwood, Holly Porter, Robin Oryem, Melissa Parker, Grace Akello

**Affiliations:** ^1^ Researcher at the London School of Economics and Political Science United Kingdom; ^2^ Professor in Practice at the London School of Economics and Political Science United Kingdom; ^3^ Professor at the London School of Economics and Political Science United Kingdom; ^4^ Director at the Social Science Research Council United States; ^5^ Researcher at the University of Kinshasa Democratic Republic of the Congo; ^6^ Researcher at the University of Gbadolite Democratic Republic of the Congo; ^7^ Independent Researcher Democratic Republic of the Congo; ^8^ Researcher at the University of Ghent Belgium; ^9^ Professor at the University of Ghent Belgium; ^10^ Independent Researcher South Sudan; ^11^ Assistant Professor at the University of East Anglia United Kingdom; ^12^ Researcher at the Centre for African Research Uganda; ^13^ Assistant Professor at the University of Cambridge United Kingdom; ^14^ Professor at the London School of Hygiene and Tropical Medicine United Kingdom

**Keywords:** Africa, Covid‐19, Democratic Republic of the Congo (DRC), governance, pandemic, public authorities, South Sudan, Uganda

## Abstract

Discussions on African responses to Covid‐19 have focused on the state and its international backers. Far less is known about a wider range of public authorities, including chiefs, humanitarians, criminal gangs, and armed groups. This paper investigates how the pandemic provided opportunities for claims to and contests over power in Uganda, the Democratic Republic of the Congo, and South Sudan. Ethnographic research is used to contend that local forms of public authority can be akin to miniature sovereigns, able to interpret dictates, policies, and advice as required. Alongside coping with existing complex protracted emergencies, many try to advance their own agendas and secure benefits. Those they seek to govern, though, do not passively accept the new normal, instead often challenging those in positions of influence. This paper assesses which of these actions and reactions will have lasting effects on local notions of statehood and argues for a public authorities lens in times of crisis.

## Introduction

On 14 February 2020, an asymptomatic Chinese national was identified as Africa's first confirmed case of Covid‐19. The second and third on 25 February were Italian nationals in Algiers, Algeria, and Lagos, Nigeria. Slightly more than a year and two waves of infections later, the World Health Organization (WHO) reported 3,111,360 confirmed cases on the continent, leading to 78,840 deaths (WHO, 2021a). Evidence was also emerging from seroprevalence surveys in Sub‐Saharan Africa that many nations may have been undercounting their infections and that the second wave was far more aggressive than the first (Usaf and Roca, 2021; Salyer et al., 2021). Concerned observers began sounding the alarm that Africa might be heading towards a devastating third wave, with only approximately seven million people vaccinated in a population of around 1.2 billion and a case fatality ratio of 3.6 per cent, as compared to the global average of between two and three per cent (WHO, 2021a).

Despite these worrying signs, Africa's Covid‐19 morbidity and mortality rates remain strikingly low when compared with those in Europe, the United States, and Latin America. There, despite many countries’ clear financial and technological advantages, a mixture of poor preparedness and political leadership have been blamed for the worst health crises since the 1918 influenza pandemic, popularly known as the Spanish flu. Africa's Covid‐19 rates are also a lot less than the continent's estimated annual rate of malaria infection of about 20 million (leading to 400,000 deaths) or the 1.1 million Africans infected with human immunodeficiency virus (HIV) (470,000 dead from related illnesses in 2018) (WHO, 2021b, 2021c). Furthermore, the death toll is just less than double the 40,000 Nigerians estimated to die every year in road traffic accidents (WHO, 2019). This highlights that while African countries’ death tolls are comparatively low so far, their capacities to deal with communicable diseases and unhealthy behaviours are extremely limited.

Commentators have variously argued that Africa benefits from a host of favourable preconditions and, less commonly, that it has found innovative ways to combat the spread of Covid‐19. The former include its young population, low rural population densities, pre‐existing cross‐reactive SARS‐CoV‐2 immunity, low levels of mobility, and low incidences of non‐communicable comorbidities (Anjorin et al., 2021; Rice et al., 2021), whereas the latter include cheap testing kits, the upscaling of cashless e‐commerce, community reporting structures, effective regional collaborations, and the repurposing of systems established to address previous health crises (Senghore et al., 2020; Smith and Crone, 2020; Travaly and Mare, 2020). However, most analysts agree that further research is needed to disaggregate the impact of such factors on the effects of the virus and to inform country‐specific solutions that address future waves (Boum, Bebell, and Bisseck, 2021).

Whatever the conclusions, Beninese economist Professor Leonard Wantchekon declared during a webinar on leadership on 2 July 2020: ‘catastrophe has been averted’ because of a combination of ‘what Africa is and what Africa has done’ (FLCA and the Institute of Global Affairs, 2020). Indeed, the pandemic has, once again, directed focus towards African governance. Much has been made of the relative speed and decisiveness with which some states responded (Pilling, 2020). Recent research based on quantitative data shows that many Sub‐Saharan African states outpaced Europeans’ efforts (Crone, 2020). For example, on average, they told people to stay at home just 13 days after the first confirmed case, whereas European states took 27 days. Sub‐Saharan African states also often imposed stricter measures, such as restrictions on movement within countries and across borders, rules on social distancing and the wearing of face masks, and the cessation of normal socioeconomic activities. African leaders, therefore, have been praised for exercising power in ways that may have made up for their countries’ significant disadvantages with respect to health systems, testing capacity, and data analytics. This, some hope, may spur a sea change in African governance styles, as leaders turn their backs on inward looking international donors that increasingly seem intent on engaging in vaccine nationalism (Lopes, 2020).

Yet, as analyses of the actions of political leaders and states continue, much less attention is being paid to what is happening on the ground, in the cities, towns, and villages that are home to the majority of Africans and those that directly govern them. A spotlight on their experiences and understanding of the pandemic may help commentators move past tired ‘Africa needs our help, again’ versus ‘Africa can teach us lessons!’ discourses (Horton, 2020). In their place, we can begin to ask what aspects of the African states’ responses to Covid‐19 are important to people's everyday lives, and what the lasting effects may be.

This is particularly vital for places already suffering from conflict, displacement, acute deprivation, health crises, and political upheaval, or where people are fearful of state officials, security forces, and possibly also international agencies. In such locations, Covid‐19 is another test of people's resilience and the leadership of those claiming positions of authority. These are the kinds of settings that the authors of this paper study, live in, and come from, and include the Democratic Republic of the Congo (DRC), South Sudan, and Uganda, all of which have long histories of wars, epidemics, and humanitarian emergencies. To varying extents their states are also unable or unwilling to govern their entire populations, let alone provide robust health systems able to confront the latest pandemic.

Sometimes termed ‘fragile or conflict‐affected states’ by the international humanitarian and development community, the Organisation for Economic Co‐operation and Development estimates that without concerted action, 80 per cent of the global poor will live in such circumstances by 2030 (OECD, 2018). For many, this means residing in places where authority and the provision of public goods may be effectively subcontracted by the government, or may in practice fall to a range of local and international actors that people understand as representing, vaguely replicating or replacing the state. Sometimes too, those exercising authority and governing people may have nothing much to do with the central state or its institutions, or they may even be actively opposed to them. They can include local traditional chiefs, self‐help groups, kinship networks, professional associations, faith‐based groups, civil society organisations, multinational companies, humanitarian agencies, security services, organised criminal gangs, militias, and rebels.

We suggest that all of these actors are ‘public authorities', which we define as ‘any kind of authority beyond the immediate family that commands a degree of consent’ (CPAID, 2018, p. 8). As they order people's everyday lives, they uphold, support, interpret, or reject prevailing social norms, and state and international policies (Lund, 2006; Hoffmann and Kirk, 2013). Successful claims to legitimate authority allow some to occupy roles as societies’ moral guardians, deciding what constitutes acceptable behaviour, who is and is not part of the community, and who does and does not deserve vital public goods such as access to health services, security, and justice. Some also use the state's weakness and crises to attempt to introduce new rules, institutions, and political orders. Such public authorities can, we argue, be akin to miniature sovereigns, able to shape people's lives and their experiences of the state in ways that have a significant impact.

To explore such dynamics, many of the paper's authors provided vignettes of life under, and public authorities’ responses to, the pandemic in the locations they know intimately.^2^ Each drew upon their existing networks and ongoing research to provide snapshots from responses to Africa's first wave of Covid‐19. Those living and working in Uganda were present for the early months of the crisis and observed as participants in their communities. They also talked with neighbours and contacts in institutions including health and education services, political parties, and the newly established response forces discussed later. Those in the DRC were already undertaking extensive fieldwork in Kongo Central and Nord Ubangi, and in Ebola‐affected areas of North Kivu, the latter in collaboration with a team of local investigators, also covering issues related to social service provision in Bukavu and Goma. The research encompassed interviews with local stakeholders, such as health workers, civil society leaders, and ordinary citizens. As the new crisis unfolded, observations were made and participants asked questions about the response measures. One researcher based in South Sudan travelled between Bor and Juba during the pandemic's initial wave, conducting related qualitative research for academic and humanitarian organisations. He contributed his observations from extensive time spent among communities facing the virus and a governance vacuum. All of the authors also followed and recorded common reactions to state broadcasts and other press and social media coverage. Researchers based across African research sites shared extracts with researchers based in Europe and the US, and research teams analysed the material together, communicating remotely via WhatsApp, conferencing software, and e‐mail. The patterns that emerge highlight the opening up of pathways and possibilities for different sorts of African public authorities and those they seek to govern.

This disruption can be seen in at least three ways across the studied countries: shifts in the balance of power between the state and other actors; contests for control of narratives and resources; and resistance to responses. Each speaks to the utility of a public authorities lens for uncovering how the pandemic is affecting people's lives, and for what may be temporary coping strategies and what may be part of or lead to long‐term changes. Through the vignettes, we are also able to see beyond the posturing and policies of national‐level leaders to how pandemic responses were experienced in places that are often written off as ungovernable or already subsumed within protracted crises. This is important for those wishing to help with the design of future responses to Covid‐19 waves and similar events, and it holds insights into the making and unmaking of statehood in places where governance is not monopolised.

The next section briefly explores the concept of, and relevant research on, public authority in Africa. We then turn to recent research from parts of Uganda, the DRC, and South Sudan where public authorities are shown to have had varying degrees of agency to enact and reinterpret state policies, yet where people have rarely accepted them uncritically. The paper concludes with a discussion of how trends identified through a public authorities lens can add to understandings of responses to the pandemic, policymaking, and statehood.

## Public authority in crises

The term ‘public authority’ has long been used to refer to instruments and organisations created by legislation to further public interests and provide public goods, such as healthcare, education, security, and justice. In Europe and the US, it has often been used in conjunction with the police, parts of the army, and various forms of, sometimes semi or fully autonomous, local administration. Indeed, there is nothing exceptional about public authority with respect to Africa in particular or developing countries in general.

Contemporary academic interest in African public authorities emerged from ethnographic literature that evaluated the micro politics of post‐colonial states. Many of its foundational authors produced research that countered depictions of them as eroding or failed polities with vast swaths of ungoverned, insecure territory. For example, Hagberg (2006) explored a charismatic leaders’ ‘making and unmaking’ of public authority in Burkina Faso; Menkhaus (2006) appraised the bargaining processes that lead to a ‘mediated state’ in areas in Sub‐Saharan Africa emerging from conflict; Olivier de Sardan (2008) examined ‘the practical norms’ of everyday governance beneath the state; Raeymaekers, Menkhaus, and Vlassenroot (2008) analysed ‘governance without government’ in situations of protracted crises; Hagmann and Péclard (2010) assessed how ‘negotiated statehood’ arises through contests over legitimacy; and Leonard and Samantar (2011) investigated the ‘local social contracts’ and ‘pro‐to‐state systems’ that can form where older regimes have retreated. Each showed how a variety of actors, from street‐level bureaucrats and security services to customary, business, and faith leaders, civil society organisations, vigilante and armed groups, claim positions of authority and, often creatively, attempt to introduce the ‘rules of the game’ that govern people by combining the provision of vital public goods with appeals to popular and emerging social norms (North, 1990).

Christian Lund (2006) was one of the first explicitly to label these authors as focused on ‘public authority'. He asserted that they were revealing how African public authorities variously seek to govern in cooperation with, alongside, in opposition to, and out of view of states. Yet, he used Abrams’ (1988) distinction between the state as a system of tangible organisations and as an idea to probe how even where they have no formal relationship with central authorities, many still draw on its symbolic repertories and mimic the practices of states. Among others, this mimicry can include taxation, the wearing of uniforms, bureaucratic procedures, and judicial‐like decision‐making. It also frequently involves purposefully blurring distinctions between state and non‐state, formal and informal, and official and unofficial through the creative blending of popular and emerging social norms—sometimes termed ‘institutional bricolage'—to introduce new modes of governance and advance public authorities’ own ends (Cleaver, 2001).

Lund (2006) suggested that this research agenda was showing how through their constant innovations and references, African public authorities ‘“bring the state back in”, but in a very different way from that described by Skocpol and others in the mid‐1980s' (Skocpol, 1985). By this he meant that the idea of the state is rarely absent from claims to, contests over, and the exercise of power, even in places where the official state's apparatus appears absent or is rejected. This acknowledges that, contrary to some of the more polemical state fragility literature, few places are untouched by experiences of the colonial or postcolonial state, and few people are opposed to some form of statehood. Nonetheless, Lund (2006) encouraged further studies of how public authority manifests across Africa rather than any concerted effort to craft a rigorous universal definition or theory.

Researchers working throughout Sub‐Saharan Africa have since documented a variety of processes that lead to public authority, with many concentrating on borderlands, conflict‐affected areas, and peripheral places. Among their many contributions, two streams of research that describe political ordering and statehood in times of crises are identifiable. The first is interested in how public authorities—sometimes with its permission or acquiescence—appropriate from central states the ability to proclaim the ‘state of exception’ as part of efforts to legitimise their power (Schmitt, 1985). Studies of vigilantes in South Africa, the governance of the Ethiopian–Somali frontier, and leaders’ appeals to the spiritual and occult in Nigeria, Sierra Leone, and Zimbabwe have suggested that a key tactic of aspiring public authorities is to define the thresholds of inclusion and exclusion with political communities, and permissions to contravene normal practices or laws, and to create new ones (Hansen and Stepputat, 2005, 2006; Buur, 2006; Hagmann and Korf, 2012). This can include declarations of who should receive public goods such as protection or a fair trial, and who can be considered as ‘bare life’ and, thereby, legitimate targets of derision, oppression, and even violence (Agamben, 1998). As part of this, public authorities often claim that they are protecting society from some spiritual, moral, or physical crisis.

Among this stream, Allen's (2015) work in northern Uganda showed how a religious leader in Gulu was able to strengthen his claims to legitimate authority by holding an ‘election’ to exile a man accused of being a vampire responsible for ongoing child murders and, therefore, a worthy subject of vigilante violence. Such actions were supported by local councillors, members of parliament, government officials, and even the police. Allen (2015, p. 2) argues that the leader engaged in a form of ‘moral populism’ that explicitly linked ‘notions of good and bad’ with formal state practices to claim that he represents ‘the will or the best interests of the people'. On a larger scale, Pendle's (2020) recent work in South Sudan describes how a female Neur prophet built a community of followers able to reject violently the central state. However, the prophet still encouraged them to make use of state‐mandated traditional courts presided over by chiefs. When they failed, she would draw on customary notions of justice to arbitrate disputes and as part of her claim to be the only authority able to wash away the ‘pollution’ caused by years of corrupt central elites’ in‐fighting.

Such research demonstrates how crises—spiritual or otherwise—can be periods within which the thresholds of inclusion become fluid, as different subjectivities—the refugee, the poor, the criminal, the unemployed, the homosexual, the mad, and other ethnic groups, inter alia—are excluded or deemed ‘bare life’ by public authorities. It also suggests that their abilities to address crises through the creative blending of social norms, informal and state practices, and the provision of public goods can be important sites of state making and unmaking in places where central authorities have not monopolised the power to govern. This, we contend, is important for understanding how routine or everyday state policies are interpreted and implemented, and what people expect from their leaders and statehood.

The second stream of research focuses on the opportunities that arise to reconfigure social order and power relations during crises, when policy responses link chains of public authorities to impart new norms and practices. It also highlights the agency and mutuality that people can cultivate to ensure that their own needs are accounted for. Here, we concentrate on the ethnographies of West Africa's Ebola epidemic of 2013–16 and the international response to it. Much has been written about how it brought local culture and foreign, often medical and bureaucratic, norms into conflict, and how the militarisation of initiatives was seemingly ignorant and, in some cases, aggressively dismissive of the people's spiritual and communal lives (Fairhead, 2016; Wilkinson et al., 2017). It has also been shown how the international response ignored or actively disrupted pre‐existing accommodations that had allowed humanitarian and local cultures to coexist.

Research on burials during the epidemic in Sierra Leone builds on these themes. Lipton (2017) describes how the response brought vast amounts of money to Freetown, including well‐paid jobs for youths who become part of official trained burial teams. However, this created tensions between what locals termed ‘black’ and ‘white’ death, with the teams gaining a reputation for being unwilling to bend strict protocols that required all bodies to be treated the same, even when this was perceived as ignoring grieving families’ wishes, oppressive or cruel. This gave rise to ‘secret burials’ undertaken in coordination with authorities such as the police, the city council, and the military that were more aligned to ‘black’ culture, while still incorporating safety practices from ‘white’ culture. Councillors in particular seized opportunities to become brokers between communities keen for a secret burial and relevant public authorities, frequently collecting large payments in the process. Lipton (2017, p. 816) argues that: ‘beneath these normative tensions was a conflict between, on the one hand, the new authorities and protocols of the state of emergency, and, on the other, established authorities, connections, and bureaucratic channels. Navigating this disjuncture became a key characteristic of living through the crisis'. The result was new, crisis‐induced, emergent forms of public authority and social order attuned to local needs and expectations.

Similarly, Parker et al.'s (2019) study of Mathaineh village outside of the capital found that locals were unwilling to call official Ebola response teams when neighbours got ill or died. Instead, a type of informal public mutuality was a guiding principle in the village, leading to collaborations on secret burials and the development of its own practices to prevent and treat Ebola, including continuously hydrating the sick before it was common among state and international responders. Nevertheless, the burials were eventually noticed by outsiders who summoned the village's chief to a meeting with public authorities connected to the state at which he was threatened with large fines. The army was also called in to ascertain that the burials had stopped, whereupon soldiers beat villagers and the chief was replaced by one appointed by the state who later died in unknown circumstances.

Alongside violations of moral and spiritual norms, the authors maintain that ‘the outbreak of Ebola exacerbated pre‐existing tensions with senior chiefdom authorities', including imparting upon villagers ‘a strong sense that “Ebola money” was passing them by’ (Parker et al., 2019, p. 448). This entrenched the villagers’ resistance to state policies and enabled the chief to legitimise further his authority by providing alternative practices. Notably, the ratio of people recovering from Ebola in Mathaineh was reportedly higher than in official treatment centres. Parker et al. (2019) conclude that crisis responders need to understand the social norms underpinning public authority in specific places when designing interventions, and that they should learn from local adaptations rather than vaguely paying lip service to notions of community engagement.

These two strands of inquiry assess how public authorities can, in part, create and shape moments of crises as they react to new policies, their followers’ expectations, and opportunities to reconfigure power relations. They also reveal that ordinary people retain agency in the face of crises and disliked responses. These themes frame the next section's exploration of vignettes from Uganda, the DRC, and South Sudan. To respect the specificities of place, they are presented by country, followed by a discussion of identifiable themes. The findings are unavoidably limited by the difficulties of conducting research during the first wave's restrictions and uncertainties. Accordingly, they should not be assumed to be generalisable or objective, and they should not be read as impressions of life at that time.

## Patronage, resistance, and love in northern Uganda

Uganda recorded its first case of Covid‐19 in March 2020. As Africa's first wave retreated in September, it had only reported 4,101 confirmed cases and 46 deaths (MoH, Uganda, 2020). President Yoweri Museveni's National Resistance Movement (NRM) government has since been praised for quickly enacting response measures such as restricting movement and commerce, locking down the national borders, clear public messaging, and the establishment of response and monitoring structures at various levels of administration (Sarki, Ezeh, and Stranges, 2020). In particular, the work of 134 District Task Forces (DTFs) set up across the country has been highlighted for identifying risks, raising awareness, social mobilisation initiatives, community dialogues, and engagement activities (WHO, 2020a).

The paper's contributing authors mainly work and live in northern Uganda, close to its borders with the DRC and South Sudan. These are places that have been the sites of conflict, resistance to President Musveni's long‐running government, and suffering from relative underdevelopment. Here, our research suggests that the pandemic response made new capital and political resources available and quickly, sometimes violently, restructured public authority. Yet, it ultimately shows that infectious disease scares and crisis politics is normal politics.

To unpack this, it helps to focus on the work of the DTFs. As in other parts of Uganda, Gulu's DTF was set up hastily and did not adhere to any clear procedural guidelines. Members were drawn from the police and security services, the district health office, municipal and district offices, and elected district councils, as well as from civil society and faith‐based organisations and the regional Acholi cultural institution, *Ker Kwaro Acholi*. The DTF has nine committees, each designed to cover aspects of the response. The resource mobilisation committee is responsible for managing Covid‐19 relief, including food that was scarce due to restrictions on movement and normal trade. Indeed, the food distribution sub‐committee beneath it had by far the largest number of attendees in records of local meetings and was particularly popular among those hoping to run in the elections of 2021. Soon there was also evidence that roles were being delegated to NRM loyalists and that its politicians were donating money and food to the DTF with the proviso that it was distributed among their constituents.

Perhaps to ensure further that pandemic relief efforts strengthened the legitimacy of central authorities and those in their networks, Museveni declared that those distributing food outside of the DTF structure would be charged with attempted murder. Opposition politicians caught doing so were arrested and beaten up. In contrast, regime politicians were allowed to distribute food to their supporters with impunity. There were also reports that non‐governmental organisation (NGO) staff— Gulu hosts missions from most of the world's large humanitarian organisations— were offering food and relief items to politicians in exchange for travel permit car stickers. The way in which Gulu's response was handled right down to the local level was, therefore, shaped by the longstanding efforts of central authorities to exert control over Uganda's northern districts. This includes the co‐optation of vital public resources that are exchanged for support and votes by networks of local public authorities (Macdonald and Owor, 2020).

The speed and aggressiveness of the response measures also took many by surprise, causing enormous economic suffering, especially among the urban poor. While Ugandans are used to and aware of the deadly potential of infectious diseases, some contrasted the restrictions with the comparatively relaxed responses to recent outbreaks of Yellow Fever, Marburg Virus, and Ebola. Added to this, many early confirmed Covid‐19 cases were among truck drivers arriving from Kenya, South Sudan, or Tanzania. For some, this was evidence that maladies such as Covid‐19 come from outside and, sometimes, that they are sent or directed with malicious intent. Ugandans with whom we spoke also feared that limits to movements between districts and regions were having the effect of ‘tribalising’ the response, entrenching older divisions between them. These boundaries were not only policed by highly militarised state actors, but also politicised militias called Local Defence Units (LDUs) (Akello, 2020). In case they were not enough, people were also encouraged to inform on one another when government guidelines were contravened.

Despite the international praise heaped on the DTFs, the state's messaging was largely delivered through long, convoluted presidential addresses broadcast on television, radio, and social media (NBS Television, 2020). People in Gulu, as in much of the world, listened closely in the hope of relaxations of the lockdown regulations, which seemed to change often without an obvious logic. Alerts were also sometimes transmitted over the radio to help security forces track down the infected. People anxiously followed them as no one wanted positive cases nearby because of fears that they would lead to all sorts of aggressive contact tracing and extra restrictions. As well, of course, it might expose them to the virus, which was quickly becoming a new struggle for people already facing challenging circumstances.

In Gulu, ground‐level public authorities—such as the controllers of boreholes and the various officials and committees that manage village jurisdictions, markets, and other daily services—were sometimes reported to be highly effective at encouraging or enforcing new practices like hand washing and mask wearing. In contrast, people noted that public authorities linked to the state had a lack of interest in containment. Rather, those charged with enforcing regulations, such as the army, police, LDUs, and elements of the health services, refashioned and continued their illicit revenue‐generating schemes. For example, as during curfews imposed in response to supposed crime waves, security forces appeared to be rounding people up at random and holding them for ransom in police station cells until their relatives paid for their release. Many understood such actions as dependent on whatever Museveni had said in his most recent announcements, with levels of brutality by security forces fluctuating based on the cover they perceived Covid‐19 policies gave them. Others presumed that there must be some political or economic benefit behind poorly explained restrictions, such as the banning of motorcycle taxis and closing of schools, although who exactly benefitted was unclear.

Similar dynamics played out in Uganda's northwest. In the early weeks, there were calls from local government officials and representatives of the Catholic Church in West Nile to provide clearer directives. Both Anglican and Catholic churches were also active in delivering public health messaging and organising congregations and the collection and distribution of tithes to the vulnerable via WhatsApp. The most popular radio stations in West Nile are funded by Christian dioceses, so they quickly became a major source of information. Perhaps for this reason, many understood Covid‐19 to be a curse from God. Islamic leaders also propelled similar explanations: ‘The first thing is Allah must be very annoyed with us because the world cannot be punished without a crime, so it's a punishment', declared a local sheik to researchers. Still, there were widespread suspicions early on, particularly among young men in the city of Arua, that lockdown measures were an act of political manipulation, or a conspiracy designed to delay the 2021 elections. These fears were heightened as there were no local Covid‐19 cases until months into the pandemic.

People with whom we spoke were also angry that the police and military beat lockdown violators and detained journalists following stories that donations received by local governments and meant for distribution through the DTF had not reached vulnerable people. Despite these harsh reprisals, some young men, particularly motorcycle taxi drivers and others whose livelihoods depend on daily labour, resisted the measures. Protests also occurred involving elders who demonstrated with bows and arrows against the establishment of an isolation centre in Arua. In the town of Moyo, the choice of location for an isolation centre angered the community to the extent that a mob burnt down the home of the district chairperson seen as responsible for commissioning its construction. People also promised to seek revenge at the ballot box.

Ongoing research on northern Ugandans’ love lives also revealed that the pandemic generated opportunities for some to challenge public authority. As in other moments when ‘normal’ ways of life are made impossible, cracks can become chasms, and spaces of manoeuvre to deviate from established norms present themselves. For instance, in usual circumstances, public authorities play a large part in governing people's romantic relationships. Young couples walking hand in hand can be beaten on the road by police; boys trying to sneak into their girlfriend's home may be beaten by her brother(s); and elders and religious leaders exert significant pressure on youths who want to circumvent their wishes and marry outside of their orbit.

Youths told us that pandemic curfews gave them excuses to stay at a girlfriend's or boyfriend's house when this would not normally be allowed. Furthermore, because of restrictions on public gatherings, some young people who would not be able to afford to wed ordinarily were able to avoid the costs of large parties, often against the wishes of religious and clan leaders and family elders. Those who bent Covid‐19 measures for their benefit had excuses backed by science, public health advice, and the militarised enforcement of the government. It was difficult for traditional authority figures to argue. Young people maintained a respectful and humble posture while ‘standing up’ to the inhibiting authority of their elders. In such ways, policies crafted in response to a crisis were unintentionally used to reconfigure intimate power relations.

## Debilitating legacies, misinformation, and violence in the DRC

The DRC's first case of Covid‐19, on 19 March 2020, was attributed to a national who had recently arrived in Kinshasa from France. By the time Africa's first wave was ebbing in September, there had been 10,536 additional cases and 271 deaths (UN OCHA, 2020). The wave overlapped with the tail end of an Ebola outbreak in the eastern provinces of Ituri and Kivu that began in late 2018 and led to around 2,280 confirmed deaths (WHO, 2020b). Both health crises were layered over a long‐running conflict in the country's eastern provinces and recent episodes of violence elsewhere. Moreover, they were met by a government with few resources and little capacity to respond, and an international humanitarian and military presence already overstretched from addressing food shortages, sanitation issues, displaced populations, and civil conflicts (Juma et al., 2020).

Customary authorities, such as chiefs and religious leaders, had a crucial role to play in the DRC's response to Covid‐19. Across the country, they raised public awareness of the virus by amplifying messages broadcast in local dialects by *griots* (town criers) and passed on regulations and protective measures issued by the central government in Kinshasa. They also threatened sanctions against recalcitrants, facilitated and led the collection of funds and in‐kind goods for the care of the sick in their communities, and protected health workers and those accused of making up infections to extract payments from attacks. In short, it quickly became apparent that without their involvement, the DRC's response would have been ineffectual.

Nevertheless, in the peaceful provincial towns of Muanda (Kongo Central) and Gbadolite (Nord Ubangi), social distancing measures were not scrupulously followed by many people. As in parts of Uganda, resistance to measures was largely due to the low number of cases. Many people, therefore, doubted the virus’ existence, with others believing that black people were immune or that the high rates of death in the West are ‘divine punishment’ for white people's immorality. These ideas were particularly acute in Muanda, where the DRC's charismatic or traditional ‘black churches’ (such as Bundu Dia Kongo/BDK, Dibundu dia Kongo dia Banduenga/DKB, and Vuvamu) spread those notions to their followers. This misinformation fed into related narratives about corruption, including suspicions about the Ministry of Health's management of Covid‐19 funds. For example, after the central government announced that it would cover the costs of treatment for people with Covid‐19, as well as the funeral costs of those who died from it, rumours spread that local health workers were ‘negotiating’ the purchase of bodies to count them as infected deaths to get more government funding.

Such incidents left customary authorities in Gbadolite and Muanda with two contradictory realities. On the one hand, they recognised that they needed to help enforce response measures decreed by the central government and, in particular, to help sensitise the population to comply with social distancing requirements that would allow them to continue cultivating fields and engaging in other livelihood activities. On the other hand, they knew that they were ultimately powerless in the face of non‐compliance by a sceptical and distrusting population. This necessitated negotiating a middle ground between raising awareness of the risks of Covid‐19 and convincing people of the dangers of a disease that many considered remote or even imaginary, and another money‐generating enterprise for the central state and its local representatives.

In the east, many of these dynamics were intensified by legacies of civil and international conflicts, as well as the heavily militarised response to the Ebola outbreak. Responses to Covid‐19 also confronted growing and frequently fierce local resistance to existing health interventions, including armed attacks on Ebola treatment centres and violence directed towards health centres, health workers, and dignified and secure burial teams. Although such incidents often seem illogical to outsiders, people in the east have long criticised the police, army, and, by association, humanitarian organisations’ disproportionate use of force when addressing Ebola (Freudenthal, 2019). They also argue that the mobilisation against the epidemic was motivated by the fear of its spread to the rest of the world, rather than a desire to treat Congolese patients. Indeed, the response was experienced as being in sharp contrast to the limited reaction to or even indifference towards other existing threats. As one local resident told the authors: ‘we die more from war than from Ebola and no one cares about it'. For many, therefore, Ebola was a business opportunity for the state and international and national NGOs. Conflicts between the Ministry of Health, international and national NGOs, and pharmaceutical companies, including the fraudulent introduction of an experimental vaccine, reinforced this suspicion (BBC, 2019). Similar statements were made by those we spoke to during Covid‐19's first wave, which people framed as a global priority that overlooked more immediate issues, including an ongoing measles pandemic and rampant insecurity.

Regardless of this challenging history, police brutality and opportunism spread quickly following the announcement of Covid‐19 response measures in Goma and Bukavu, the respective capitals of the eastern DRC's North and South Kivu provinces. As part of the declared state of emergency, provincial governors established police commissions to enforce the requirements. Members included health ministers, representatives of the police, army, and intelligence services, and the hospitals selected to treat Covid‐19 patients. For local residents, the commissions’ most noticeable actions were stopping and fining those without face masks, often using roadblocks staffed by officers and soldiers who circulated throughout the cities. The fines were confusing, not properly communicated, and therefore not respected. Indeed, their implementation and amount depended on who you were and who you knew. In Bukavu, police also worked with taxi drivers and intelligence services to create a shortage of public transport in anticipation of an 8:00 pm curfew. They then induced traffic jams, which prevented people from getting home, allowing them to be stopped, pulled from vehicles, and fined or arrested. At one field site, which lies on the DRC–Uganda border, close to Kasese, women were even beaten for attempting to cross a river to cultivate their crops on their land on the DRC side; soldiers were said to have sought money or sexual favours in return for turning a blind eye to unauthorised crossings.

In response, residents in both provinces occasionally rose up against such abuses. The vast majority of this resistance was spontaneous, consisting of angry people supporting one another against perceived police overreach as it occurred. Nonetheless, there were more organised episodes. For example, the citizens of Essence—one of Bukavu's poorest neighbourhoods, where people depend on mobility for their livelihoods—refused to remain in their houses and the police had no option but to flee the area. Youths also organised themselves to protest outside the city's Bwindi treatment centre after a young taxi driver was killed by police for not wearing a mask on 15 June. The boy was popular in his neighbourhood so that night youths took to the streets, protesting and burning tyres until police fired bullets to disperse them. The next morning, they blocked roads in Bagira commune while another group of youths went on to attack the Bwindi treatment centre.

Our research suggests, however, that this was not the full story. Those with whom we spoke linked the violence to a conflict between actors involved in the province's Covid‐19 response, specifically Governor Théo Kasi Ngwabidje, Dr Denis Mukwege, and members of the Ministry of Health. They had jointly decided to designate three hospitals for the treatment of Covid‐19 patients: Provincial Reference Hospital; Panzi Hospital; and Saint Luc Hospital. A few days later, though, Ngwabidje unilaterally took the decision to set up a centre in Bwindi for the care of the sick, thereby sidelining these hospitals. In retaliation, Dr Mukwege publicly denounced a lack of transparency in the management of funds allocated to the province's pandemic response by the central government, and then resigned from his position, apparently in an effort to safeguard his honour and that of Panzi Hospital, which he had founded. His resignation added to the population's doubts about the official response to the epidemic and a growing perception that for some it was the latest business in town. A few days later his rival's centre was attacked.

The multiple causes of local resistance to the response are difficult to unpack. Yet, there is a negotiated line in the places where the authors work. Residents are accustomed to managing low‐level extortion on the part of a predatory police force, which is usually proportional to the perceived socioeconomic standing of the targeted individual. Arrests or trips to police stations, for actual or fabricated infractions, are avoided through contributions of ‘beans for the children’ or ‘water'—small sums of money determined by the guilty party—to irregularly paid state officials. In exchange, the police are expected to let individuals go and to respond to their calls in cases of robbery or unrest in neighbourhoods. But our research pointed towards a growing feeling that the police and others had used the pandemic measures to cross this line, breaking carefully negotiated social norms and practices, and running roughshod over the consent to the police's fragile authority that they engender. In response, some neighbourhoods in Bukavu and Goma started community watches or hired military personnel to fulfil the role of unwelcome Covid‐19‐enforcing officers. In these ways, abuses and mistrust led to, albeit temporarily, pockets of public mutuality.

## Political stagnation and violent competition in South Sudan

As in the DRC, South Sudan faced the dilemmas and the potential devastation of Covid‐19's first wave whilst dealing with a pre‐existing protracted complex crisis, including a civil war that began in 2013, a collapsed oil‐dependent economy, and the absence of uniform public services. To make matters worse, however, it did so with a vacuum of state and local government. In February 2020, President Salva Kiir had issued a presidential decree removing all of the governors, state ministers, and commissioners. This was justified as a necessary prerequisite for establishing the Revitalized Transitional Government of National Unity that was promised by South Sudan's 2018 peace deal and looking more likely due to recent agreements between the young country's warring parties.

Many South Sudanese with whom we spoke wanted the government to appoint directly the states’ leaderships. They argued that the vacuum was already causing problems, such as exacerbating the long‐running conflict between the Lou Nuer and Murlei in Jonglei state, as well as impeding a proper response to the virus in many areas. In their stead, the national Ministry of Health in collaboration with the World Health Organization (WHO) took the lead in disseminating information on the virus and outlining restrictions in mid‐March that banned social gatherings, schooling, religious services, and political rallies. This was followed by a night‐time curfew a few weeks later, which effectively halted some humanitarian organisations’ work; many international staff left South Sudan.

In Jonglei, trusted chiefs with long histories as powerful interlocutors between the government, aid agencies, and communities were thrust to the forefront of response efforts. They travelled from community to community, meeting with elders, church leaders, and women's groups to explain that the virus is real and to attend to people's growing despair. They also sought to counter distrust of foreigners, including United Nations workers, who were quickly associated with Covid‐19, with some calling it ‘the Kawajas’ [white people] virus’ or the ‘town people virus'. Some chiefs carried buckets and soap to demonstrate good hygiene practices to communities. In addition, to address a feared lockdown‐induced secondary food crisis, they encouraged community members to share whatever they have with needy neighbours and, in some cases, provided money to the most desperate that contacted them through elders.

There was another side‐effect of the first wave: emerging anecdotal evidence suggested that the lockdown was also contributing to a rise in petty crime, such as house break‐ins and shop and cattle theft. Furthermore, organised youth cattle raiding across county borders was believed to have increased as a result of the heightened competition for resources due to the pandemic response measures. It is unclear whether or not chiefs were involved in sanctioning this. Regardless, they are mandated by the Transitional Constitution of the Republic of South Sudan and the Local Government Act, 2009, to handle petty crime cases in their courts. But the lockdown posed additional difficulties to the summoning of alleged perpetrators to account for themselves. Moreover, the vacuum meant some were also having to arbitrate more significant disputes to prevent episodes of violence. Thus, as Covid‐19 cases rose during the first wave, chiefs found themselves caught between their normal duties, the need to interpret and implement response measures, and to prevent more serious social unrest.

## Crisis, responses, and statehood

This paper has explored research from specific places within Uganda, the DRC, and South Sudan during Africa's first wave of Covid‐19. We adopted a public authorities lens that focused on how pandemic response policies were perceived and interpreted at the local level. It revealed how some actors used the crisis to claim legitimacy to govern others and advance their own ends. We also found that some people took the opportunity to challenge the status quo or to reject the new normal, often by contesting poor leadership or engaging in forms of public mutuality. Although the studied contexts are unique, three key patterns emerge across them:

- First, the pandemic responses fed into and exacerbated shifts in the balance of power between public authorities connected to the state and those with more ambiguous relationships. In Uganda, the government sought to consolidate its overall control and, in the process, to deny opportunities to those outside of its networks. With notable exceptions, the situation in the DRC was slightly more collaborative, with religious leaders lending their legitimacy to messaging by distrusted state and international actors. In contrast, in South Sudan, the pandemic struck at a moment of political vacuum, leaving traditional chiefs to take the lead in responding. Recent histories of upheaval, including the legacies of previous health crises, shaped these mobilisations and collaborations in ways that meant policies were rarely simply transplanted from plans to practice. This afforded some public authorities opportunities to strengthen their claims to power by calming anxieties, imparting knowledge, organising resources, and addressing the pandemic's side‐effects.
- Second, contests for control over pandemic narratives and resources led public authorities to proclaim who was to blame, deserving of relief, or a potential target of sanctions. In Uganda, the central state directed support to its followers, while its poor messaging created fears about the external and potential internal sources of the virus. It also gave security services opportunities to abuse their powers. Similar issues arose in the DRC where an outbreak of Ebola had already engendered distrust of foreign‐endorsed militarised health interventions. And there was widespread scepticism of the ability of authorities connected to the state to police fairly the pandemic or to handle transparently vital resources, with violent backlashes in some places. In the absence of the state or a unified narrative, respondents suggested that South Sudan's other public authorities may have intensified their violent competition for resources.
- Third, in parts of Uganda and the DRC, these dynamics created resistance to pandemic responses and challenges to the status quo. Although this was mostly confined to grumblings and rumours concerning their true purpose, in some places resistance was more tangible and organised, even resulting in an attack on a state official's property. The lockdown measures also gave young couples the opportunity to challenge rules that had long governed courtship. In the eastern DRC resistance was more overt. Here, security services that had crossed unwritten lines were called out and even ejected from the places that they were meant to serve. In some instances, this led to communities taking matters into their own hands and policing the pandemic themselves. Yet, many still suspected hidden hands were at play.

Our research suggests that we should not uncritically subscribe to narratives that either praise or dismiss Africa's response to the first wave of Covid‐19. To understand how pandemic policies were implemented, it is necessary to look at the role of public authorities, especially in places where the state must compete for or does not monopolise authority. This has enabled us to show how response policies may have been declared with little capacity or intention to enact them transparently or fairly. We have also revealed how those responsible for doing so on the ground viewed them as opportunities or burdens, with actors having varying degrees of agency to interpret them as they see fit. This made some akin to miniature sovereigns, able to shape people's experiences of the pandemic and, by extension, the state in significant ways.

Where this was predominantly one of power accumulation and abuse, it is likely that authorities’ future responses to crises will be met with scepticism and rejections. So too may their efforts to define their visions of statehood. But where public authorities and communities were able to collaborate with the state or to carve out pockets of public mutuality to police themselves and provide alternatives to the status quo, lessons should be learnt. Further research on such instances could be vital for those interested in how pandemic narratives, resources, and local innovations can be better harnessed to address crises, especially where they require cooperation among public authorities with differing histories and levels of legitimacy to effect change in challenging settings.

## Acknowledgements

Thanks to all contributors who worked during a trying time to collect data and document life under the pandemic and responses to it. Unless otherwise stated, this paper was principally funded by the Economic and Social Research Council (grant number: ES/P008038/1) and conducted at the Centre for Public Authority and International Development, London School of Economics and Political Science. Liz Storer's research was funded by a Knowledge Frontiers grant from the British Academy, and Melissa Parker and Grace Akello's research on Covid‐19 and public authority is co‐supported by funding from the Pandemic Preparedness project of the Wellcome Trust (Grant number: NH/17033).

## Data availability statement

The data that support the findings of this study are available on request from the corresponding author. The data are not publicly available due to privacy or ethical restrictions.

## Endnotes

2 Longer versions of the vignettes are available in a working paper by Green and Kirk (2020).
